# The Effect of Brushing and Post-Processing Procedures on the Optical Properties of Printed Dental Resin

**DOI:** 10.3390/polym18121521

**Published:** 2026-06-18

**Authors:** Roxana Diana Vasiliu, Liliana Porojan, Flavia Roxana Bejan, Sorin Daniel Porojan, Anamaria Matichescu

**Affiliations:** 1 Department of Dental Prostheses Technology (Dental Technology), Center for Advanced Technologies in Dental Prosthodontics, Faculty of Dental Medicine, “Victor Babes” University of Medicine and Pharmacy, Eftimie Murgu Sq. No. 2, 300041 Timisoara, Romania; roxana.vasiliu@umft.ro (R.D.V.); flavia.toma@umft.ro (F.R.B.); 2 Department of Oral Rehabilitation (Dental Technology), “Victor Babes” University of Medicine and Pharmacy, Eftimie Murgu Sq. No. 2, 300041 Timisoara, Romania; porojan.sorin@umft.ro; 3 Department of Preventive, Community Dentistry and Oral Health, Center for Advanced Technologies in Dental Prosthodontics, Faculty of Dental Medicine, “Victor Babes” University of Medicine and Pharmacy, Eftimie Murgu Sq. No. 2, 300041 Timisoara, Romania; matichescu.anamaria@umft.ro

**Keywords:** dental polymers, WID, whiteness, polymerisation, activated charcoal, thermal activation, glycerin barrier, optical stability

## Abstract

The optical stability and aesthetic performance of dental polymers are critical for the longevity of restorative treatments. This study aims to evaluate the influence of different polymerisation conditions and maintenance protocols—specifically the use of charcoal pastes and brushes—on the Whiteness Index for Dentistry (WID) of polymer samples. Four groups of polymer samples (Optiprint Lumina, Dentona, AG) were prepared under various conditions: Group I (Standard: 7 min, 22 °C), Group II (Thermal: 7 min, 60 °C), Group III (Extended: 20 min, 22 °C), and Group IV (Glycerin barrier: 7 min, 22 °C). The protocol consisted of brushing with standard or carbon-infused toothpastes and brushes for a period of 2 min. Between brushing, the samples were stored in artificial saliva at 37 °C to simulate the oral environment. The samples were divided into groups. The first group was brushed with a standard paste and a standard brush; the second group was brushed with a simple paste and a carbon brush; the third group was brushed with carbon paste and a standard brush; and the fourth group was brushed with carbon paste and a carbon brush. Changes in the Whiteness Index for Dentistry (WID) were recorded and analysed statistically using a paired *t*-test and Pearson correlation. Results: All groups showed a statistically significant increase in the WID (*p* = 0.0191). Group IV (glycerin barrier + carbon paste/brush) exhibited the highest increase, with a WID = 2.0, demonstrating a synergistic effect between oxygen inhibition control and activated charcoal. In contrast, Group II (thermal polymerisation) showed the highest chromatic stability (ΔWID = 0.6), remaining below the Whiteness Perceptibility Threshold (WPT = 0.72). No significant correlation was found between polymerisation time and WID changes (r = 0.087, *p* = 0.913), indicating that temperature and surface treatment are the primary drivers of optical modification. Conclusions: The heat parameter did not reveal a significant difference in the optical properties. Furthermore, the combination of a glycerin barrier and carbon-based hygiene products maximises the whitening effect while remaining within the Whiteness Acceptability Threshold (WAT < 2.60), providing valuable insights for both material processing and patient maintenance protocols.

## 1. Introduction

Additive manufacturing, also known as 3D printing, has gained significant prominence in dentistry, evolving from use in diagnostic models to functional restorations [1]. In particular, 3D-printed resin restorations have received considerable attention [2]. The first 3D-printed resins contained little or no filler material and were primarily used for temporary restorations due to concerns about long-term durability, biocompatibility, and mechanical properties [3]. Recent advancements in resin formulations, technologies, and post-processing have addressed many of these issues, enabling the production of restorations that meet the requirements of both temporary and permanent applications.

Vat polymerisation remains the primary manufacturing process. Digital Light Processing (DLP) enables rapid production by simultaneously curing entire resin layers using a DLP projector or a digital micromirror device that emits ultraviolet light [4,5]. This process is particularly suitable for high-volume applications, including the fabrication of dental aligners, temporary and permanent restorations, and other objects. 3D-printable materials exhibit considerable heterogeneity, with significant differences in mechanical and physical properties. Fillers have been used to enhance resin properties, particularly in permanent restorative materials rather than temporary ones [3]. Despite improvements in the mechanical properties of 3D-printed resin restorations, challenges remain in colour stability, water sorption, solubility, and anisotropy. Three-dimensional printing resins are validated for temporary and midterm use for up to 2 years, and are suitable for various types of restorations [4,5].

The properties of 3D-printed restorations depend on factors at each production stage: pre-printing (resin composition), printing (hardware, orientation, layer thickness), and post-processing (curing, rinsing, polishing). These factors directly influence the degree of conversion, mechanical strength, and biocompatibility of the final restoration. The clinical performance, longevity, and acceptance of 3D-printed restorative materials in mainstream dentistry are constrained by several limiting factors. 3D-printed restorations offer benefits including digital efficiency, parallel production, and reduced waste [1]. Post-processing is essential for transforming a 3D-printed object from its pre-polymerised state into a functional final restoration. Cleaning, curing, and finishing directly affect the mechanical strength, aesthetic properties, and biocompatibility of the final product. Cleaning usually involves rinsing with isopropyl alcohol (IPA) to remove unpolymerised resin. IPA is flammable and presents health risks similar to ethanol. Alcohol exposure may also cause surface discolouration in filled materials by dissolving surface monomers. Some manufacturers now recommend centrifugal cleaning, which increases the degree of conversion (DC) and reduces surface roughness without using solvents [6]. Longer washing times and higher concentrations further reduce residual monomers, thereby improving the degree of conversion and the flexural strength of printed objects [1]. Post-curing is necessary to enhance the mechanical strength and biocompatibility of 3D-printed parts. This process depends on factors such as curing chamber type, UV light wavelength and intensity, temperature, exposure duration, and resin composition [7,8]. It is also known that the post-polymerisation process facilitates the conversion of residual unreacted monomers in the outer layers [8,9,10]. Despite manufacturers’ guidelines for resins, limited scientific information remains regarding the optimal printing processes and post-curing techniques to achieve restorations with adequate mechanical and aesthetic properties when using 3D-printed resins and equipment [11]. Regarding aesthetic performance, 3D-printed resins exhibit considerable variability across shades and show limited colour stability after water storage [9]. Furthermore, prolonged exposure to violet light emitted by the post-curing unit may alter the final colour of the restoration. Another critical parameter to evaluate is the surface smoothness of provisional restorations, as this characteristic is essential for preventing biofilm accumulation, preserving the colour of final restorations, and maintaining periodontal tissue health.

In recent years, research on tooth colour has expanded substantially, and numerous studies employing diverse methodologies have been conducted to enhance understanding of the susceptibility of dental hard tissues to staining and the efficacy of bleaching agents [12,13]. This growing interest reflects the impact of tooth whiteness on individuals’ appearance and social interactions, as well as a trend toward preferring whitened teeth over natural tooth colour [14]. Tooth discolouration can have various aetiologies, but it is primarily due to the consumption of coloured beverages and foods, which are frequently part of daily dietary habits. The location and the intensity of the discolouration can vary widely [15]. As noted previously, 3D-printed resins, owing to their intrinsic properties, can exhibit colour changes, even when used for provisional restorations and with limited time in the mouth.

Manufacturers continually reformulate whitening toothpastes, using a wide range of physical and chemical ingredients to achieve a synergistic whitening effect. Physically, abrasive agents, such as silica, remove extrinsic stains through abrasion [16,17,18]. Dentifrices comprise a category of products that includes toothpastes, gels, and powders. These products are primarily used to improve oral health. These pastes are suitable for brushing onto enamel and temporary restorations. Additionally, they are the most widely used vehicles for delivering active agents. To enhance whitening, hydrogen peroxide, commonly used in professional treatments, can be incorporated into toothpaste at lower concentrations [16].

Activated charcoal has recently gained popularity, particularly in oral care products. Charcoal has been incorporated into various oral care products [17]. Activated charcoal is produced by heating carbon-based materials with agents that create a porous internal structure. This activation process yields a material with high adsorption capacity. Adsorption is the process by which molecules adhere to and remain on the surface of a solid material [18,19,20,21,22,23].

This study aimed to compare various post-polymerisation protocols for dental resins in conjunction with four distinct brushing methods, each using different toothpastes and brushes.

The null hypotheses were formulated as follows:

**H0.**  *Colour change will be similar across all samples when comparing alternate cycles of staining and brushing, which simulate clinical conditions, with staining followed by extended brushing.*

**H1.**  *Whitening toothpastes will not differ from regular toothpaste in terms of overall tooth colour change related to the whitening index (WID).*

**H2.**  *There will be a difference in the optical parameters after polishing with the two toothpastes included in this study.*

**H3.**  *There will be a difference in the optical properties related to the different brushing techniques.*

## 2. Materials and Methods

### 2.1. Preparation of 3D Printed Samples

The sample size was calculated using G*Power (version 3.0.10). Under the assumption of a normal distribution and an effect size of 0.49, the required sample size was determined for α = 0.05 and a statistical power of 0.90. Sixty-four samples were prepared (*n* = 8). In in vitro dental research on colour stability, highly concentrated staining solutions, such as complex artificial broth, and active whitening protocols typically produce pronounced differences between groups. According to Cohen’s guidelines and established dental biomaterials literature employing similar Student *t*-test designs, a large effect size of 0.40 is both standard and clinically relevant for detecting meaningful experimental changes in optical properties without unnecessarily increasing sample sizes.

**Figure 1:**
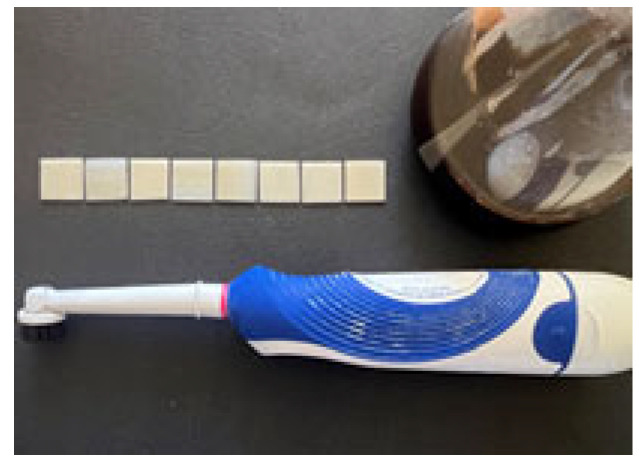
The samples from the printed resin and the setting for the experiment.

Rectangular samples (10 × 10 mm) were printed from an additive resin (Optiprint Lumina, Dortmund, Germany) with a thickness of 1 mm. The specimens were designed in CAD software (Asiga Composer 2.0., Sydney, Australia) and printed with a digital light processing printer (Asiga UV Max, Sydney, Australia) using the specified parameters. For all samples, the layer thickness was standardised to 50 µm, and the sample was positioned on the platform at 90°. Silicon carbide paper was used to gradually polish the specimens to 2000 grit. The samples were post-polymerised according to the selected protocol, and after that were stored in distilled water at room temperature prior to testing. The samples are presented in Figure 1.

### 2.2. Post-Processing Procedures

After printing, the specimens were washed with isopropyl alcohol for 2 min and post-cured in a UV light unit (BB-Midi Plus, Mecatronics, Trento, Italy) for varying periods. The samples were divided into four groups based on their UV treatment, as follows:

- The UV polymerisation lasted 7 min at 22 °C.
- The UV polymerisation took place for 7 min at 60 °C.
- The UV polymerisation took place for 20 min at 22 °C
- The UV polymerisation took place for 7 min at 22 °C, and a layer of glycerin was applied before polymerisation.

### 2.3. Brushing Procedures

Brushing was performed with an electric toothbrush (Oral-B, Procter & Gamble, Boston, MA, USA). The samples were brushed 28 times for 2 min with a force stroke of 2.5 N using different slurry pastes and brushes: a standard brush and a charcoal brush (Oral-B, Procter & Gamble, Boston, MA, USA). The samples were brushed for 2 min after immersion in the staining colourant for 5 min at 37 degrees Celsius, then rinsed in distilled water. To produce the slurry pastes, two types of toothpastes were selected: a standard paste (Colgate Total Original, New York, NY, USA) and a whitening paste (Colgate Max White Charcoal, New York, NY, USA) (Table 1). A total of 1 g of distilled water was combined with 2 g of toothpaste.

The first group was brushed with a standard paste and a standard brush; the second group was brushed with a simple paste and a carbon brush; the third group was brushed with carbon paste and a standard brush; and the fourth group was brushed with carbon paste and a carbon brush. Between brushings, the samples were immersed in artificial saliva and maintained at 37 °C. The materials included in this study are presented in Table 1.

### 2.4. Staining Treatment

The staining broth was prepared by combining 100 g of finely ground instant coffee (Nescafé, Nestlé Coffee Brand, Vevey, Switzerland), 50 g of black tea (Black Tea, English Breakfast, Twinings, Hampshire, UK), 0.125 g methylparaben, 0.075 g propylparaben, 533 g red wine (Caii de la Letea, Cabernet Sauvignon, Bucharest, Romania), 2 g mucine, and 1.5 mL yellow (Knightsbridge PME Ltd., Enfield, UK) and red colourants (Knightsbridge PME Ltd., Enfield, UK), all disolved in 1 L of ultrapure water.

The specimens were immersed in the staining solution under constant agitation at 37 °C for 28 days. After each staining step, the brushing protocol was applied to every sample. After staining, a new colour measurement (T1) was performed and compared with the initial values (T0), using the formula (CIEDE2000).

### 2.5. Colour Measurements

The spectrophotometer was calibrated before measurement, and the CIELAB colour scale (L*, a*, b*) was measured using a spectrophotometer (Vita EasyShade, Vita Zahnfabrik, Bad Säckingen, Germany) under D65 illuminant. All samples were registered before and after by the same operator to ensure standardisation. The samples were registered against white and black backgrounds using a specialised card (Whibal, Picture Flow LLC, San Anselmo, CA, USA).

Translucency, opalescence, contrast ratio, and whiteness index were calculated using the following formulas. Four measurements were performed at the top of the specimens and in the centre of the samples.

Translucency parameter [24]:

TP = {(L_B_* − L_W_*) ^2^ + (a_B_* − a_W_*)^2^ + (b_B_* − b_W_*)^2^} ^½^,

where B and W represent the values of specimens on the black and white backgrounds.

To standardise colour change measurements for clinical relevance, ΔE* values were converted to National Bureau of Standards (NBS) units using the following formula [25,26]:

NBS = ΔE* × 0.92.

Colour change levels, measured in NBS units, are defined as follows: 0.0–0.5 indicates an extremely slight change, 0.5–1.5 a slight change, 1.5–3.0 a perceivable change, 3.0–6.0 a marked change, 6.0–12.0 an extremely marked change, and 12.0 or more indicates a change to another colour.

The whiteness index in dentistry (WID) and the white index difference (ΔWID) were calculated to quantify the degree of whiteness of the materials [27].

WID = 0.511 × L* − 2.324 × a* − 1.1 × b*;

ΔWID = WID2 − WID1.

WID differences were determined by calculating the mathematical difference between the values recorded at a given stage and those of the control group for the same material. Thresholds for colour evaluations serve as essential quality control measures. The perceptibility threshold (WPT) and acceptability threshold (WAT) were set at 0.72 and 2.60 WID units, respectively [28].

The opalescence parameter (OP) was calculated using the following formula [29]:

OP =√[(a_B_* − a_W_*)^2^ + (b_B_* − b_W_*)^2^]

where B and W represent the values of specimens on the black and white backgrounds.

### 2.6. Statistical Analysis

All calculated and measured data are presented as means ± standard deviations. Differences between materials were evaluated using an unpaired Student’s *t*-test for each stage. Paired Student’s *t*-tests compared values before and after wear [29]. Regression analysis and Pearson’s correlation assessed relationships between variables across phases. Pearson’s coefficient (r) was interpreted as follows: 0 to 0.2 (very weak), 0.2 to 0.4 (weak), 0.4 to 0.6 (moderate), 0.6 to 0.8 (strong), and 0.8 to 1.0 (very strong). A significance level of 0.05 was used. The *p*-value was chosen *p* < 0.05.

## 3. Results

### 3.1. Translucency Parameter (TP)

**Figure 2:**
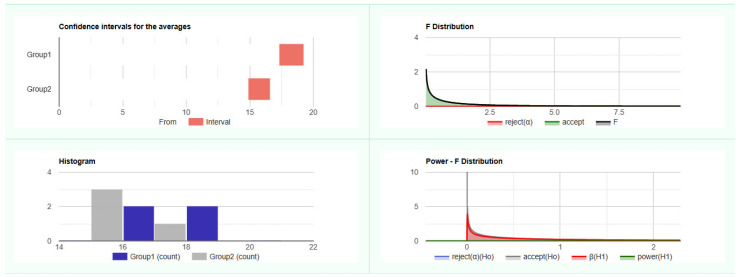
Statistical distribution among the groups of the translucency parameter before and after colouring, and different pastes and brushes.

**Figure 3:**
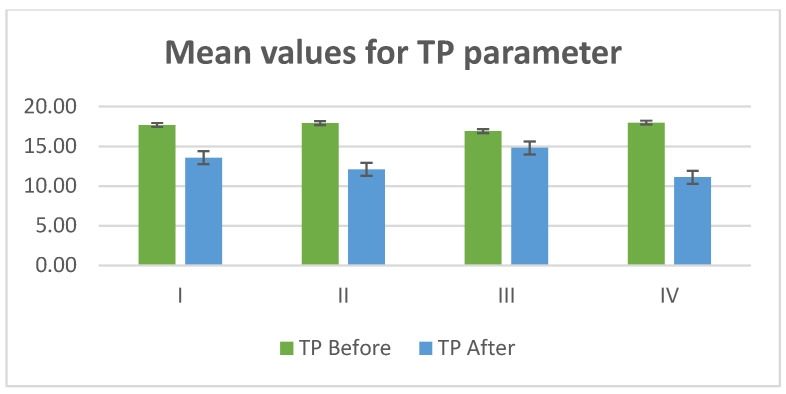
Mean values for the translucency parameter before and after colouring, and different pastes and brushes.

The TP values of the printed samples are presented in Table 2, Table 3 and Table 4. Graphical representation of the TP values is shown in Figure 2 and Figure 3. The TP values differed significantly between groups (*p* < 0.05). There were significant changes before and after colouring and brushing for all tested samples.

Among the post-polymerisation types, the third group, which was post-polymerised the most, exhibited the lowest value due to increased b* values, resulting in a yellowish appearance. Statistical analysis revealed that the temperature difference applied during the post-polymerisation process did not have a significant effect (*p* > 0.05).

Regarding the various pastes and brushes, there were significant differences between the groups. The samples brushed with carbon paste and a carbon brush exhibited the lowest TP values after treatment. The third group registered the highest TP values.

Significant differences were found between the first and fourth groups (*p* < 0.05) and between the third and fourth groups (*p* < 0.05).

Group IV exhibited a loss of −6.88 units (38.2%), indicating that this combination was the most aggressive. The interaction between carbon-infused bristles and abrasive carbon paste resulted in severe surface wear, thereby increasing light scattering and substantially reducing translucency. The comparison between Group I (−4.11) and Group II (−5.80) demonstrated that replacing a standard brush with a carbon brush significantly increased optical degradation. These findings indicated that carbon-infused filaments are inherently more abrasive to 3D-printed polymers than standard nylon bristles. Group III exhibited the highest resistance to translucency loss (18.3%). This outcome suggests that using a standard brush reduced the abrasiveness of carbon particles in the paste compared to carbon-on-carbon brushing. Statistically significant changes were reported between the values registered before and after brushing and staining (*p* < 0.05).

### 3.2. Opalescence Parameter (OP)

**Figure 4:**
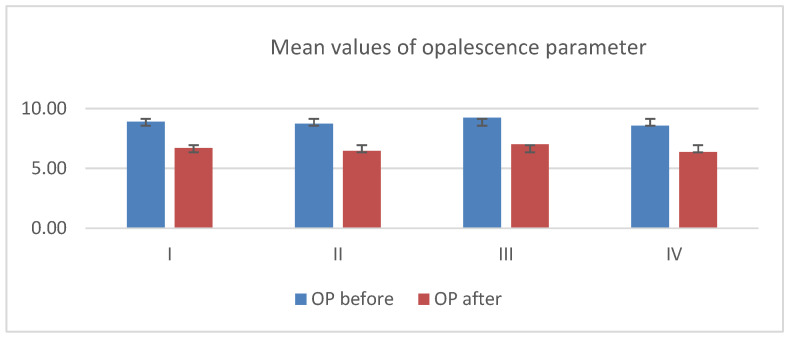
Mean values for the opalescence parameter before and after colouring and different brushing methods.

The opalescence parameter was calculated using the previous formula and the L*a*b* parameters. The OP values are presented in Table 5 and Figure 4. Printed resin samples demonstrated high initial opalescence (mean 8.90), indicating strong biomimetic aesthetic potential. Combined staining and carbon fibre abrasive brushing reduced the OP. Although the residual value of 5.70 remained acceptable for dental restorations, the loss of opalescence is accompanied by surface matting and reduced specular reflections, underscoring the importance of glazing to protect surface texture.

### 3.3. Contrast Ratio Parameter

**Figure 5:**
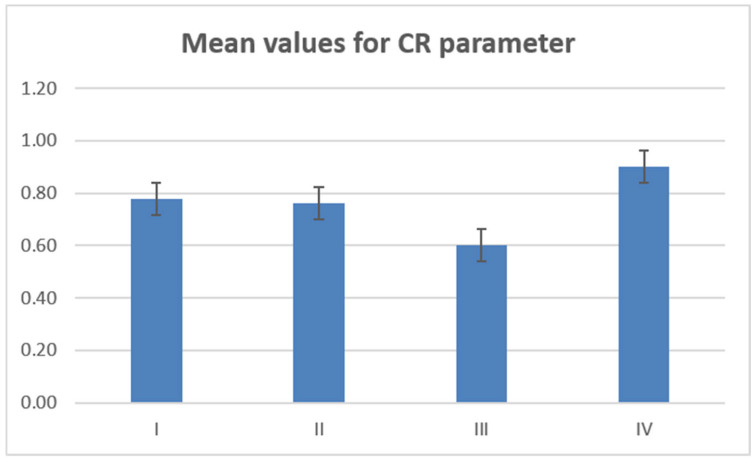
Mean values for the contrast ratio parameter calculated after the different post-polymerisation protocols, colouring, and brushing.

The contrast ratio (CR) values identified in this study range from 0.60 to 0.90, indicating a consistent tendency for resin-printed samples to become opaque under abrasive conditions. The highest observed value of 0.90 occurred in group IV—the group brushed with carbon paste and a carbon brush—indicating an almost complete loss of translucency, suggesting significant surface degradation (Figure 5). Statistical differences between the groups are shown in Table 6.

### 3.4. Whitening Index

**Figure 6:**
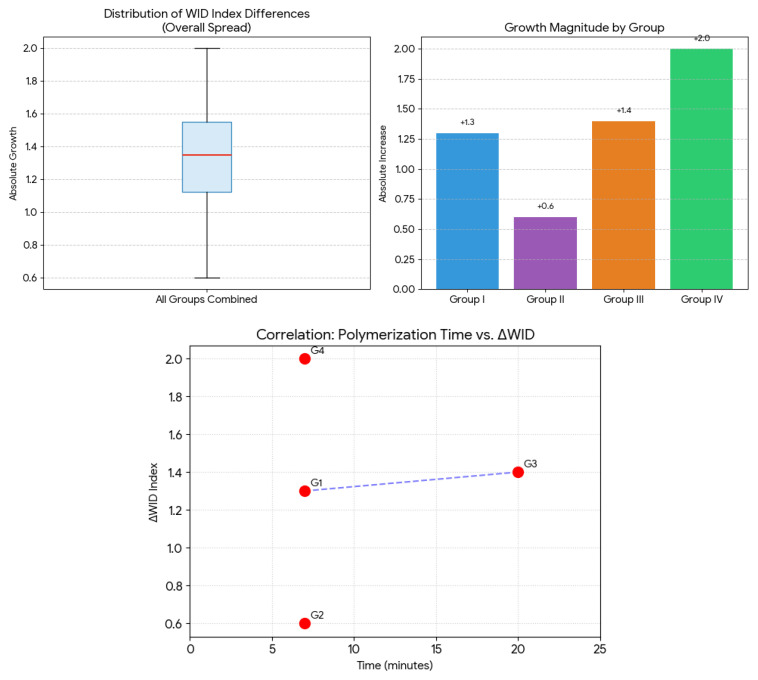
Mean values for the WID parameter related to colouring and brushing of the samples.

Mean values of the whitening index (WID) are shown in Table 7 and Figure 6. The values increased across all groups, but more notably for groups III and IV, which used carbon paste. Groups III and IV benefited the most from the protocol, exceeding the unity threshold, a value considered significant in dental aesthetics for validating the effectiveness of a cleaning treatment. Groups III and IV used carbon paste in the protocol. Student’s *t*-test revealed a significant difference between group IV and group III (*p* < 0.005).

### 3.5. Colour Change (∆E)

Regarding colour stability, Table 8 shows that group IV exhibited a perceptible difference. Results were organised by clinical significance. Group IV showed the highest colour variation, classified as “Clearly a perceptible difference”. This indicates that the material in this group was susceptible to staining or significant changes in shade after the experimental protocol. Groups I and II were categorised as “Perceptible, but acceptable”. Although the colour change was visible, it remained within clinical thresholds and does not require replacement of the dental material. Group III demonstrated the best colour stability, with a change classified as “Slightly perceptible”, indicating that it is the least affected by staining and brushing among the groups.

## 4. Discussion

The first (H0) and second (H1) null hypotheses were rejected; the printed samples exhibited different optical properties across the specified parameters. In this study, a printed dental resin for temporary restorations was evaluated after exposure to various post-processing protocols, colouring, and brushing with different pastes and brushes (Table 9).

The second and third hypotheses were accepted. The two techniques generate distinct surface micro-textures, resulting in different interactions between light and the specimens. Group IV produces a specific micro-topography that alters the reflection of light at the boundary layer. This modification changes the ratio of specular reflection (surface gloss) to diffuse reflection (scattered light) in a manner distinct from Group III. Concerning aesthetic preservation, the interaction between bristle and paste alters the Translucency Parameter (TP) and final colour stability metrics differently across groups. These findings indicate that optical degradation during daily hygiene is influenced not only by the toothpaste but also by the specific combination of bristle and paste. In the present study, the samples were printed vertically with a layer thickness of 50 microns. Several studies have shown a link between surface roughness and colour stability with increased layer thickness. The study found that there were no significant differences between 25 and 50 microns, but there were between 50 and 100 microns. Increasing layer thickness increases surface roughness and affects colour stability due to a higher rate of staining [30]. Further investigations should be made to investigate this aspect.

The colour values were measured with a spectrophotometer, and L*, a*, and b* were calculated. In the literature, there are no studies on this type of resin (Optiprint Lumina, Dentona, Dortmund, Germany) using this specific post-processing and whitening protocol. As stated by the manufacturer, this resin is a photopolymerised resin with high filler content. The inorganic filler contains nanofillers that contribute to a higher lustre and, therefore, to more stable optical properties, such as the whitening index [11]. In this study, different values were recorded after post-processing protocols. The heat parameter did not reveal a significant difference. The primary factor affecting optical properties was the longer UV curing time. Post-curing is an essential step in the 3D printing workflow because it improves the material’s properties.

In the literature, several studies contradict each other regarding the post-curing effect on optical properties. While post-curing is a critical phase in the 3D printing workflow for optimising material properties, findings in two similar studies indicate that variations in post-curing duration did not yield significant differences in the Translucency Parameter (TP) across any thickness groups. Several articles have studied this type of print with respect to its optical properties. The article concluded that Optiprint Lumina mimicked the glowing appearance of natural enamel under varying atmospheric light or UV rays much better than traditional materials [31]. Another article [32] confirmed that Optiprint Lumina’s inorganic-filled matrix guarantees high material stability. However, surface treatments and systematic polishing are required. Brushing causes minor surface alterations that change the light refraction profile, meaning a smooth mechanical polish is mandatory to lock in long-term colour stability. These results suggest that minor adjustments in post-curing protocols might be insufficient to trigger measurable changes in optical properties. This lack of significance points to a potential ‘plateau effect’, in which optical stabilisation occurs early in the polymerisation process. Consequently, further research would yield more meaningful results. Nevertheless, the indispensable role of post-curing in enhancing other essential characteristics, such as mechanical strength and long-term stability, remains a primary consideration for clinical success [33,34]. Another study in the literature demonstrates that post-curing time directly affects optical parameters. Exposure longer than that recommended by the manufacturer results in decreased TP and increased yellowing (b*) [35]. Similar findings were observed in this study: the third group exhibited lower translucency parameters before and after colouring and brushing compared with the other groups. Another study in the literature that focuses on printed resins shows that extended polymerisation improves mechanical stability but reduces translucency, thereby compromising aesthetics due to increased polymer density [36]. Another similar study concluded that there is a “sweet spot”: under-polymerisation leaves the material too translucent and unstable, while prolonged polymerisation increases opacity [7]. Staining frequently occurs in all restorative materials after extended use. Changes in the optical properties have been attributed to oxidation of the polymer matrix or oxidation of unreacted double bonds in the residual monomers [37].

There was a difference in the optical parameters after the polishing with the two toothpastes included in this study. Distinct shifts in optical parameter values were recorded between the two toothpaste formulations, confirming that the abrasive/adsorptive properties of the charcoal paste alter the polymer’s optical profile.

The third and fourth hypotheses were accepted. The synergy between the carbon-infused brush and carbon paste (Group IV) produced a unique optical outcome compared to using a standard brush with the same paste (Group III). Intense staining, combined with prolonged mechanical brushing, leads to synergistic degradation of the optical properties of 3D resins. Mechanical brushing disrupts the dye layer, resulting in an aged, opaque appearance [38,39]. Simultaneous application of a brush and a paste containing carbon particles accelerated the degradation of optical properties, resulting in a greater reduction in translucency (TP) than conventional methods. This effect is attributed to increased surface roughness and enhanced mechanical retention of abrasive particles. A strong correlation was identified between reduced translucency and diminished opalescence. Group IV exhibited the greatest decline in both parameters (TP loss of 38.2% and OP loss of 37.4%), indicating that the combined abrasive action of carbon-infused brushes and pastes results in substantial degradation of the polymer’s optical network. In contrast, Group III exhibited the highest optical stability, suggesting that standard nylon brushes may reduce the abrasive effects of carbon-based dentifrices and help preserve the material’s aesthetic properties.

A few clinical indications for patients with printed dental prostheses can be drawn from this research. Given that all samples were printed vertically at a 90-degree angle and subjected to different curing and brushing protocols, the following clinical guidelines are recommended. To maintain the colour stability of the restorations (Group III), the samples should undergo a longer UV light treatment of at least 20 min. This can help the material set more effectively, seal the layers, and make the restorations more resistant to external stains. Another aspect that was observed concerned using charcoal toothpaste with charcoal brushes (Group IV), which produced the largest and most noticeable colour change. Dentists should tell patients that these products are very abrasive and can scratch the resin’s surface, revealing the layers of the 3D print and increasing the likelihood of staining over time. Regarding the toothbrush, patients should use a regular toothbrush with soft or medium bristles, and toothpaste that does not contain charcoal and is not too abrasive [40]. As shown in Group I, regular cleaning methods keep colour changes within acceptable limits.

The limitations of this study include the fact that only one type of printed resin was tested and only a few whitening pastes were evaluated. Further research should focus on other printed resins and different whitening pastes.

## 5. Conclusions

Within the limitations of this in vitro study, the following conclusions were drawn:

1. The primary factor affecting optical properties was the longer UV curing time.
2. The heat parameter did not reveal a significant difference on the optical properties.
3. The best combination for preserving optical properties is carbon paste and a soft, simple brush.
4. The combination of carbon paste and brush leads to lower optical properties due to increased surface roughness.

## Figures and Tables

**Table 1:** Materials included in this study and their composition.

Name of the Product	Manufacturer	General Composition	Active Abrasive Agents
Colgate Total Original	Colgate-Palmolive, São Bernardo do Campo, SP, Brazil	Water, Glycerin, Hydrated Silica, Sodium Lauryl Sulfate, Aroma, Cellulose Gum, Poloxamer 407, Tetrasodium Pyrophosphate, Xanthan Gum, Benzyl Alcohol, Cocamidopropyl Betaine, Sodium Saccharin, Phosphoric Acid, Sucralose, CI 77891	Hydrated silica, calcium pyrophosphate
Colgate Max White Charcoal	Colgate-Palmolive, São Bernardo do Campo, SP, Brazil	Water, Sorbitol, Hydrated Silica, PEG-12, Tetrapotassium Pyrophosphate, Sodium Lauryl Sulfate, Aroma, Potassium Hydroxide, Cellulose Gum, Phosphoric Acid, Cocamidopropyl Betaine, Sodium Fluoride, Sodium Saccharin, Xanthan Gum, Charcoal Powder, Limonene	Sodium fluoride, hydrated silica, charcoal powder
Oral B Pro Series	Procter & Gamble (P&G), Marktheidenfeld, Germany		
Optiprint Lumina,	Dentona AG, Dortmund, Germany	Methacrylate Resins • Inorganic Fillers • Photoinitiators • Colour Pigments • Stabilisers	

**Table 2:** Statistically significant changes between the groups.

Source	DF	Sum of Square	Mean Square	F Statistic	*p*-Value
Groups (between groups)	1	13.1072	13.1072	38.5176	0.000807
Error (within groups)	6	2.0417	0.3403		
Total	7	15.1489	2.1641		

**Table 3:** Statistical analysis of the translucency parameter between the groups.

Experimental Groups	TP Before Treatment (Mean ± SD)	TP After Treatment (Mean ± SD)	Mean Loss (Units)	Percentage Loss (%)	Clinical Degradation Tier
Group I (Standard Brush)	17.67 ± 0.01	13.56 ± 0.02	−4.11	23.30%	Moderate degradation
Group II (Carbon Brush)	17.90 ± 0.02	12.10 ± 0.01	−5.8	32.40%	Severe degradation
Group III (Standard Brush + Carbon Paste)	16.90 ± 0.01	13.80 ± 0.03	−3.1	18.30%	Highest resistance to loss
Group IV (Carbon Brush + Carbon Paste)	17.98 ± 0.03	11.10 ± 0.02	−6.88	38.30%	Most aggressive protocol

**Table 4:** Statistical analysis of the translucency parameter between the groups.

Source of Variation	Sum of Squares (SS)	Degrees of Freedom (df)	Mean Square (MS)	F-Statistic	*p*-Value	Statistical Significance
Between Groups	13.1072	1	13.1072	38.5176	0.000807	Highly Significant (*p* < 0.001)
Within Groups (Error)	2.0417	6	0.3403			
Total	15.1489	7	2.1641			

**Table 5:** Mean and standard deviation (SD) values for the opalescence parameter before and after colouring and brushing.

Groups	OP Before	OP After
I	8.90 ± 0.01	5.70 ± 0.02
II	8.72 ± 0.02	6.47 ± 0.02
III	9.22 ± 0.02	8.02 ± 0.02
IV	8.57 ± 0.02	5.37 ± 0.02

**Table 6:** Statistically significant differences between the samples related to the contrast ratio (CR) parameter.

Comparison of Optical Property Values Between Groups	*p*-Value for TP	*p*-Value for CR	*p*-Value for OP
I–II	0.55	0.45	0.56
I–III	0.46	0.05	0.45
I–IV	0.05	0.05	0.65
II–III	0.07	0.04	0.52
II–IV	0.35	0.05	0.26
III–IV	0.04	0.04	0.52

**Table 7:** Mean values for the WID parameter related to colouring and brushing of the samples.

Groups	WID—Before	WID—After
I	17.4	18.7
II	17.6	18.2
III	16.4	17.8
IV	17.5	19.5

**Table 8:** Mean values for the ∆E parameter after colouring and brushing protocols.

Groups	(∆E)	Clinical Interpretation
I	2.2	Perceptible, but acceptable
II	2	Perceptible, but acceptable
III	1.9	Slightly perceptible
IV	3.2	Clearly a perceptible difference

**Table 9:** Studied hypotheses in this research.

Hypothesis	Status	Statistical/Clinical Justification
H0: Colour change will be similar across all samples when comparing alternate cycles of staining and brushing, which simulate clinical conditions, with staining followed by extended brushing.	Rejected	The (*p*)-value of (0.0191) (*p* < 0.05) demonstrates that the variations in all the optical parameters are not uniform and depend significantly on the specific polymerisation and brushing protocols.
H1: Whitening toothpastes will not differ from regular toothpaste in overall tooth colour change, as measured by the whitening index (WID).	Rejected	The charcoal-based toothpaste (Group IV, (ΔWID = 2.0)) significantly outperformed the standard toothpaste in altering the dental whiteness index.
H2: There will be a difference in the optical parameters after the polishing with the two toothpastes included in this study.	Accepted	Distinct shifts in optical parameter values were recorded between the two toothpaste formulations, confirming that the abrasive/adsorptive properties of the charcoal paste alter the polymer’s optical profile.
H3: There will be a difference in the optical properties related to the different brushing techniques.	Accepted	The synergy between the carbon-infused brush and carbon paste (Group IV) produced a unique optical outcome compared to using a standard brush with the same paste (Group III).

## Data Availability

The original contributions presented in this study are included in the article. Further inquiries can be directed to the corresponding author.

## References

[1] Keßler A., Montenbruck L., Schwendicke F., Lüchtenborg J., Kaisarly D. (2025). Narrative review of 3D-printed temporary and permanent dental resin restorations. Polym. Test..

[2] Yüceer Ö.M., Kaynak Öztürk E., Çiçek E.S., Aktaş N., Bankoğlu Güngör M. (2025). Three-Dimensional-Printed Photopolymer Resin Materials: A Narrative Review on Their Production Techniques and Applications in Dentistry. Polymers.

[3] Alshamrani A., Alhotan A., Kelly E., Ellakwa A. (2023). Mechanical and Biocompatibility Properties of 3D-Printed Dental Resin Reinforced with Glass Silica and Zirconia Nanoparticles: In Vitro Study. Polymers.

[4] Huh J., Moon Y.-W., Park J., Atala A., Yoo J.J., Lee S.J. (2021). Combinations of Photoinitiator and UV Absorber for Cell-Based Digital Light Processing (DLP) Bioprinting. Biofabrication.

[5] Kuang X., Yue L., Qi H.J., Zhou K. (2023). Introduction to 4D Printing: Concepts and Material Systems. Additive Manufacturing Technology.

[6] Mayer J., Reymus M., Wiedenmann F., Edelhoff D., Hickel R., Stawarczyk B. (2021). Temporary 3D printed fixed dental prosthesis materials: Impact of post printing cleaning methods on degree of conversion as well as surface and mechanical properties. Int. J. Prosthodont.

[7] Kim D., Shim J.S., Lee D., Shin S.H., Nam N.E., Park K.H., Shim J.S., Kim J.E. (2020). Effects of post-curing time on the mechanical and color properties of three-dimensional printed crown and bridge materials. Polymers.

[8] Bayarsaikhan E., Lim J.H., Shin S.H., Park K.H., Park Y.B., Lee J.H., Kim J.E. (2021). Effects of postcuring temperature on the mechanical properties and biocompatibility of three-dimensional printed dental resin material. Polymers.

[9] Reymus M., Fabritius R., Keßler A., Hickel R., Edelhoff D., Stawarczyk B. (2020). Fracture load of 3D-printed fixed dental prostheses compared with milled and conventionally fabricated ones: The impact of resin material, build direction, post-curing, and artificial aging-an in vitro study. Clin. Oral Investig..

[10] Alharbi N., Osman R.B., Wismeijer D. (2016). Factors influencing the dimensional accuracy of 3D-printed full-coverage dental restorations using stereolithography technology. Int. J. Prosthodont..

[11] Kessler A., Hickel R., Reymus M. (2020). 3D printing in dentistry-state of the art. Oper. Dent..

[12] Revilla-León M., Meyers M.J., Zandinejad A., Özcan M. (2019). A review on chemical composition, mechanical properties, and manufacturing work flow of additively manufactured current polymers for interim dental restorations. J. Esthet. Restor. Dent..

[13] Chen H., Cheng D.H., Huang S.C., Lin Y.M. (2021). Comparison of flexural properties and cytotoxicity of interim materials printed from mono-LCD and DLP 3D printers. J. Prosthet. Dent..

[14] Oskui S.M., Diamante G., Liao C., Shi W., Gan J., Schlenk D., Grover W.H. (2015). Assessing and reducing the toxicity of 3D-printed parts. Environ. Sci. Technol. Lett..

[15] Park S.H., Lee C.S. (1996). The difference in degree of conversion between light-cured and additional heat-cured composites. Oper. Dent..

[16] Scotti C.K., Velo M.M.A.C., Rizzante F.A.P., Nascimento T.R.L., Mondelli R.F.L., Bombonatti J.F.S. (2020). Physical and surface properties of a 3D-printed composite resin for a digital workflow. J. Prosthet. Dent..

[17] Pontefract H., Courtney M., Smith S., Newcombe R.G., Addy M. (2004). Development of methods to enhance extrinsic tooth discoloration for comparison of toothpastes. 1. Studies in vitro. J. Clin. Periodontol..

[18] Kwon S.R., Wertz P.W. (2015). Review of the mechanism of tooth whitening. J. Esthet. Restor. Dent..

[19] Newton J.T., Subramanian S.S., Westland S., Gupta A.K., Luo W., Joiner A. (2021). The impact of tooth colour on the perceptions of age and social judgements. J. Dent..

[20] Moldovan A.M., Popescu V., Ionescu C.V., Cuc S., Craciun A., Moldovan M., Dudea D., Mesaros A.S. (2022). Various aspects involved in the study of tooth bleaching procedure: A questionnaire-based study. Int. J. Environ. Res. Public Health.

[21] Gupta A., Gallagher J.E., Chestnutt I.G., Godson J. (2021). Formulation and fluoride content of dentifrices: A review of current patterns. Br. Dent. J..

[22] Brooks J.K., Bashirelahi N., Reynolds M.A. (2017). Charcoal and charcoal-based dentifrices: A literature review. J. Am. Dent. Assoc..

[23] Naidu A.S., Bennani V., Brunton J.M.A.P., Brunton P. (2020). Over-the-counter tooth whitening agents: A review of literature. Braz. Dent. J..

[24] Wang J., Yang J., Lv K., Zhang H., Huang H., Jiang X. (2023). Can we use the translucency parameter to predict the CAD/CAM ceramic restoration aesthetic?. Dent. Mater..

[25] Shirani M., Savabi O., Mosharraf R., Akhavankhaleghi M., Hebibkhodaei M., Isler S. (2021). Comparison of translucency and opalescence among different dental monolithic ceramics. J. Prosthet. Dent..

[26] Paravina R., Powers J. (2004). Esthetic Color Training in Dentistry.

[27] Guerra F., Mazur M., Corridore D., Pasqualotto D., Nardi G.M., Ottolenghi L. (2015). Evaluation of the esthetic properties of developmental defects of enamel: A spectrophotometric clinical study. Sci. World J..

[28] Pérez M.M., Herrera L.J., Carrillo F., Pecho O.E., Dudea D., Gasparik C., Ghinea R., Bona A.D. (2019). Whiteness difference thresholds in dentistry. Dent. Mater..

[29] Al-Fodeh R.S., Al-Dwairi Z.N., Almasri M., Baba N.Z. (2024). Mechanical properties of 3D-printed resin denture teeth: An in vitro study. J. Prosthodont..

[30] Ziyad T.A., Abu-Naba’a L.A., Almohammed S.N. (2021). Optical properties of CAD-CAM monolithic systems compared: Three multi-layered zirconia and one lithium disilicate system. Heliyon.

[31] Astudillo-Rubio D., Delgado-Gaete A., Bellot-Arcís C., Montiel-Company J.M., Pascual-Moscardó A., Almerich-Silla J.M. (2018). Mechanical properties of provisional dental materials: A systematic review and meta-analysis. PLoS ONE.

[32] Luo W., Westland S., Ellwood R., Pretty I., Cheung V. (2009). Development of a whiteness index for dentistry. J. Dent..

[33] Espinar C., Della Bona A., Pérez M.M., Pulgar R. (2022). Color and optical properties of 3D printing restorative polymer-based materials: A scoping review. J. Esthet. Restor. Dent..

[34] Sasany R., Jamjoon F.Z., Kendirci M.Y., Yilmaz B. (2024). Effect of Printing Layer Thickness on Optical Properties and Surface Roughness of 3D-Printed Resins: An In Vitro Study. Int. J. Prosthodont..

[35] Yay Kuscu H.Y., Gorus Z. (2025). Translucency, color, and hardness of 3D-printed provisional restorations. Sci. Rep..

[36] Lee E.H., Ahn J.S., Lim Y.J., Kwon H.B., Kim M.J. (2022). Effect of post-curing time on the color stability and related properties of a tooth-colored 3D-printed resin material. J. Mech. Behav. Biomed. Mater..

[37] Lee H.J., Cho S.H., Kim C., Mai H.N., Win T.T., Rana S., Lee D.H. (2025). Effects of Post-Curing Duration on Color Changes in 3D-Printed Fixed Polymeric Restorations of Varying Thicknesses. Int. J. Prosthodont..

[38] Bezgin T., Özer L., Tulga Öz F., Özkan P. (2015). Effect of toothbrushing on color changes of esthetic restorative materials. J. Esthet. Restor. Dent..

[39] Tahayeri A., Morgan M., Fugolin A.P., Bompolaki D., Athirasala A., Pfeifer C.S., Ferracane J.L., Bertassoni L.E. (2018). 3D printed versus conventionally cured provisional crown and bridge dental materials. Dent. Mater..

[40] Alharbi N., Alharbi S., Cuijpers V.M., Osman R.B., Wismeijer D. (2018). Three-dimensional evaluation of marginal and internal fit of 3D-printed interim restorations fabricated on different finish line designs. J. Prosthodont. Res..

